# Advancing the Assessment and Treatment of Comorbid Pediatric Chronic Functional Abdominal Pain (CFAP) and Restrictive Eating Disorders

**DOI:** 10.3390/children10091539

**Published:** 2023-09-11

**Authors:** Emily A. Beckmann, Claire M. Aarnio-Peterson, Kristen E. Jastrowski Mano

**Affiliations:** 1 Department of Psychology, University of Cincinnati, Cincinnati, OH 45221, USA; beckmaea@mail.uc.edu; 2 Division of Behavioral Medicine and Clinical Psychology, Cincinnati Children’s Hospital Medical Center, Cincinnati, OH 45229, USA; claire.aarnio-peterson@cchmc.org

**Keywords:** eating disorders, adolescents, anorexia nervosa, chronic functional abdominal pain, functional gastrointestinal disorders, chronic pain

## Abstract

The aim of this review is to heighten awareness of the association between chronic functional abdominal pain (CFAP) and restrictive eating disorders (ED) in adolescents. We describe current diagnostic practices and propose future research efforts to improve the assessment and treatment of comorbid CFAP and restrictive EDs. A narrative review of the literature on CFAP and EDs was performed using *PubMed*, *JSTOR*, *ScienceDirect*, and *PsycINFO* and the following search terms: ‘restrictive eating disorders’, ‘chronic functional abdominal pain’, ‘chronic pain’ ‘treatment’ ‘diagnosis’ and ‘adolescents’. Published studies on restrictive EDs and CFAP from May 2008 to March 2023 were included. Ascribable to the overlap in etiology and symptom presentation, adolescents with chronic pain are significantly less likely to have their ED pathology promptly identified by providers compared to adolescents without comorbid chronic pain. This highlights the importance of the time sensitive and accurate identification of EDs in adolescents with CFAP. Overall, assessment methods are limited and EDs take longer to be identified in adolescents with comorbid CFAP. Future efforts should address diagnostic practices in pediatric settings and improve the communication among medical and mental health providers in order to promote the rapid and effective diagnosis and treatment of comorbid CFAP and EDs.

## 1. Introduction

Chronic Functional Abdominal Pain (CFAP) is a highly prevalent functional gastrointestinal disorder (FGID) characterized by abdominal pain occurring most days over the course of at least three months [1]. CFAP is the most common cause of long-term abdominal pain among adolescents, with upwards of 17.9% of adolescents in the United States receiving the diagnosis [2]. To date, CFAP does not have a clear pathophysiological explanation, such that there tend to be few if any abnormal laboratory findings or imaging results to explain the patient’s pain [1]. CPAP is not associated with intestinal motility, differentiating it from other disorders of gut-brain interaction or FGIDs, such as irritable bowel syndrome, abdominal migraine, and functional dyspepsia [1]. Psychosocial, environmental, and genetic factors [1,2] contribute to CFAP. Several brain regions have been linked to pain [3,4,5,6,7]. A subcortical circuit in the brain has also been associated with the subconscious processing of pain-related emotions, such that when this circuit becomes dysregulated, pain-related anxiety and rumination can develop in response [7]. Repeated injury to the abdomen may also put youth at higher risk for developing CFAP [8]. For example, multiple surgeries and/or infections in the same abdominal region could impact the sensitivity of nerve receptors in the gut [9]. Similarly, central sensitization—when the central nervous system undergoes changes that alter its processing of pain and other sensory stimuli, resulting in a lower pain threshold [9,10] is recognized as a contributor to the development and maintenance of chronic pain [11,12,13]. Abdominal pain may lead to food avoidance and dietary restriction, which places individuals at higher risk for developing an eating disorder pathology [13]. Moreover, pediatric chronic pain is conceptualized using a biopsychosocial model, whereby biological mechanisms involved in the onset and maintenance of abdominal pain are considered, as well as the child’s cognitions, emotions, behaviors. and sociocultural factors [10,14,15]. Restrictive eating disorders (EDs) are diagnosed in approximately 3.8% of females and 1.5% of males in the United States [16]. Restrictive EDs involve self-imposed dietary restriction, malnutrition, and weight loss [16]. *Restriction* is a marked characteristic of several DSM-5 [16] feeding and eating disorders; however, it is most often associated with anorexia nervosa (AN), atypical anorexia nervosa (AAN), and avoidant/restrictive food intake disorder (ARFID). AN is characterized by caloric restriction and an intense fear of weight gain, leading to significantly low body weight in the context of development, sex, and/or physical health; AN also has the highest mortality rate of any psychiatric condition [17,18]. Temperament, environment, genetics, and physiology have been linked to the development of AN [16,17,18,19,20]. Children and adolescents with immediate family members with AN are at increased risk for developing disorder [19]. Moreover, a range of neurological abnormalities and gastrointestinal symptoms have been linked to AN [21,22]; however, efforts are still being made to parse out the abnormalities associated with the disorder versus the effects of malnutrition. It is important to highlight that the historical and cross-cultural variability in the prevalence of AN bolsters its association with cultures and environments where thinness is valued [23].

If full criteria for AN is met but the adolescent’s weight is still within or above the normal range, a diagnosis of AAN is made [16]. The etiology of AAN is thought to be similar to factors indicated in youth with AN but remains relatively understudied. However, restriction in individuals who are at or above the normal weight range often experience the same degree of physical and psychological impairment as underweight individuals [20,24].

ARFID is another restrictive ED characterized by malnutrition, reflected by significant weight loss, nutritional deficiency, and interference with psychosocial functioning as a result of an eating or feeding disturbance (e.g., lack of interest in eating, avoidance of food for sensory purposes) [16]. ARFID differs from AN in that dietary restriction occurs in the absence of distress related to shape, weight, and figure. From an etiological perspective, several factors have been linked to the development of ARFID. First, genetic and physiological characteristics such as a history of gastrointestinal conditions, as well as other medical problems in infancy and childhood have been associated with the development of ARFID [16,25].

Diagnosing CFAP and/or restrictive EDs is challenging given their overlapping symptom presentations. The accurate identification of EDs in adolescents with comorbid chronic pain is significantly delayed compared to adolescents without chronic pain [13]. A hampered diagnosis is particularly troubling given the prognostic importance of early detection of EDs in treatment outcomes [26]. Similarly, despite a dearth of research on the diagnosis of CFAP among adolescents with EDs, the importance of the early identification and treatment of pain symptoms in adolescents with EDs is clear as it could serve as a barrier to treatment or lead to the development of a more severe pain condition [13].

The aim of this review is to heighten awareness of the association between CFAP and restrictive eating behaviors. We highlight current diagnostic practices and propose future research efforts to improve the diagnosis and treatment of comorbid CFAP and restrictive EDs. We argue for the development of (1) standardized assessment protocols to support pediatric physicians and mental health professionals in the differential diagnosis of CFAP and/or restrictive Eds, and (2) evidence-based interdisciplinary approaches to assessment and treatment.

## 2. Method

This review aims to provide an overview of published studies on restrictive EDs, CFAP, and chronic pain from May 2008 to March 2023. To review the literature, we searched for articles using *PubMed*, *JSTOR*, *ScienceDirect*, and *PsycINFO*, using search terms including the following: ‘restrictive eating disorders’, ‘chronic functional abdominal pain’, ‘chronic pain’ ‘treatment’ ‘diagnosis’ and ‘adolescents’. Studies were excluded if: (1) they were not published in English, (2) the target population was children and adolescents with *binge eating disorder* or *bulimia nervosa*, or (3) the target population was children and adolescents with *acute pain*. Evaluation of the literature involved review of articles by the first author and two pediatric psychologists (with expertise in pediatric chronic pain and EDs) for content related to the subject area.

## 3. Restrictive EDs in Adolescents with Chronic Functional Abdominal Pain (CFAP)

CFAP may put an individual at risk for the development of a restrictive ED. Diet, emesis, and nutritional status associated with CFAP can have an impact on nutrition and weight, which in turn could contribute to the development of a restrictive ED [13]. Patients with GI disease often have diets lower in calories and greater evidence of malnutrition compared to healthy controls [27]. Dietary restriction and malnourishment often suppress appetite, promote preoccupation with food, and negatively impact mood, attention, concentration, and sleep [28,29,30,31,32]. Dietary restriction in response to abdominal pain is common, often as a means to avoid discomfort. Interestingly, temporal relationships between pain and ED symptoms have been found in other pain populations as well [33,34]. For example, among adult low-back pain patients, the onset of disrupted eating behavior set in after pain chronification [33]. The successive psychological and behavioral changes that occur in response to dietary restriction could impact youth with CFAP who restrict their diet in response to pain [35,36]. Dietary restriction in response to abdominal pain is not uncommon and could take place for a variety of reasons. Sim and colleagues [13] suggest that chronic abdominal pain might promote dietary restriction in response to an individual’s avoidance of discomfort. Beyond fear of pain and avoidance of discomfort, social reinforcement in response to weight loss may further reinforce and/or promote dietary restriction among adolescents with chronic pain [13]. 

In the converse direction, adolescents with restrictive EDs may go on to develop CFAP symptoms. Sim and colleagues [13] suggest equivalent rates of adolescents presenting with chronic pain prior to the onset of an eating disorder (41.2%) and adolescents presenting with an eating disorder prior to developing chronic pain (35.3%). Gastrointestinal distress is common among adolescents with restrictive EDs [13], with upwards of 25% of adolescents with restrictive eating experiencing gastrointestinal discomfort and pain [27]. Pain complaints are related to changes in food intake, lack of variety in diet, and the consequential impacts on gastric motility [27]. 

In summary, it may often be unclear whether restrictive EDs precede or coincide with the duration of a chronic pain condition, such as CFAP [13]. However, research suggests that regardless of presentation order, chronic pain and EDs impact one another.

### Shared Vulnerabilities and Risk Factors

It is possible that as a result of shared vulnerabilities and risk factors, adolescents with CFAP are at increased risk for developing restrictive eating and vice versa. Individuals with EDs and chronic pain possess shared temperamental risk factors, such as tendencies toward perfectionism [37] and harm avoidance [38,39]. Both groups have also demonstrated an increased risk for other psychiatric disorders, such as anxiety and depression [40,41]. More recently, it has been suggested that central sensitization—a recognized risk factor in the development and maintenance of chronic pain [9,11,12,13]—may also play a role in eating disorders [42].

Adverse childhood experiences, such as emotional abuse from caregivers and financial instability, have been reported as risk factors for the development of chronic pain and EDs [43,44]. Social factors also increase the likelihood of developing eating disorder pathology in adolescents with chronic pain [23,28,29]. The social reinforcement of weight loss, in particular, is linked to an increased frequency of restrictive eating behaviors to maintain low weight [16]. The interrelatedness of restrictive EDs and CFAP symptoms among adolescents is notable. Thus, the accurate detection and diagnosis of their co-occurrence is critical. 

## 4. Current Assessment Practices for CFAP and Restrictive EDs

Both CFAP and EDs are diagnosed by means of a physical exam and psychological assessment. It is common for youth experiencing chronic abdominal pain as well as youth with restrictive eating to initially present to their pediatrician [45], a gastrointestinal specialist [46], or family medicine practitioner [47]. Physicians determine the duration of the pain, physical abnormalities, history of disease, as well as weight loss. If alarm features (e.g., gastrointestinal bleeding) are *not* present, a physician might suspect CFAP [48].

The psychological assessment for CFAP utilizes a biopsychosocial framework [14,15,49]. If assessed within an interdisciplinary context, pediatricians and psychologists work together to assess the child to collect medical, psychological, and social history from the child, parents/caregivers, teachers, and other providers. Clinicians also inquire about cognitive, school, physical, and sleep impairment [14,15,49,50].

The psychological assessment for EDs focuses on the patient’s weight concerns, body image, and eating disorder behaviors (e.g., excessive exercise, caloric restriction in efforts to control weight) [13,22]. There are a variety of tools that have been developed for eating disorder screening in medicine [51,52,53,54,55,56]. For example, SCOFF [51] uses the following questions to determine if a patient might meet diagnostic criteria of AN or Bulimia Nervosa (BN): (1) Do you make yourself **S**ick because you feel uncomfortably full? (2) Do you worry that you have lost **C**ontrol over how much you eat? (3) Have you recently lost more than **O**ne stone (equivalent to 14 pounds/6.4 kg) in a 3-month period? (4) Do you believe yourself to be **F**at when others say you are too thin? (5) Would you say that **F**ood dominates your life? If a patient answers “yes” to two or more of the five questions, the physician should follow up with a comprehensive diagnostic evaluation. The Eating Disorder Diagnostic Scale (EDDS) [52], a self-report ED symptom measure and the Eating Disorder Examination (EDE) [53], an interview based on DSM-5 criteria are also commonly used in medical settings.

Comorbid CFAP and restrictive EDs can be difficult to diagnose as many physical symptoms overlap [13]. Both conditions can involve weight fluctuation, food refusal, fasting, fatigue, orthostatic intolerance, early satiety, reflux, and/or constipation [16,57]. Moreover, behavioral symptoms such as restricting certain food groups or foods as well as skipping meals are both behaviors seen in CFAP and restrictive eating disorders [13].

In addition to the innate complexity in diagnosing a restrictive ED in the context of co-occurring pain symptoms, other barriers to comprehensive assessment include limited appointment times in primary care and a lack of available screening and assessment tools [35,54].

## 5. Recommendations for Assessment of CFAP and Restrictive EDs

### 5.1. Current Challenges

Restrictive EDs among adolescents presenting with chronic pain are likely under-diagnosed [13,22]. Though restriction and other shared risk factors between CFAP and eating disorder symptoms should be considered by clinicians when a patient presents with a potential comorbidity, utilizing existing ED screeners (such as SCOFF [51], described above) is likely not the best solution.

Previous studies have found that eating disorder assessments—involving questions focused on intentional dietary restriction, body image, and distress surrounding weight, shape, and figure [51,52]—could be *un*helpful in the context of CFAP, as pediatric chronic pain patients tend to primarily focus on somatic symptoms (e.g., abdominal pain, nausea) [58,59]. Therefore, adolescents with CFAP might inadvertently underreport weight, shape, and body image concerns. Furthermore, eating disorder symptom screeners developed for use in primary care settings have limited psychometric evidence in pediatric populations [51,52,54,55,56]. Finally, it has been demonstrated that tools for identifying AN in a general population have low specificity in the identification of eating disorders in patients with existing gastrointestinal complaints [57,60].

It may take additional effort to disentangle some of the common behaviors reported in both groups. For example, skipping meals may be reported by patients and/or caregivers undergoing assessments for CFAP or restrictive EDs. However, pain may offer a socially acceptable excuse for food avoidance [61]. Pain related complaints and/or stating that one “is not hungry” in response to abdominal pain could maintain restrictive eating and weight loss under the guise of pain/discomfort, delaying necessary psychological care for an ED. Development of a restrictive ED in the context of CFAP could exacerbate or explain comorbid physical symptoms associated with chronic pain.

### 5.2. Improve Identification of Comorbidity through Standardized Screening and Assessment Protocols

**Figure 1:**
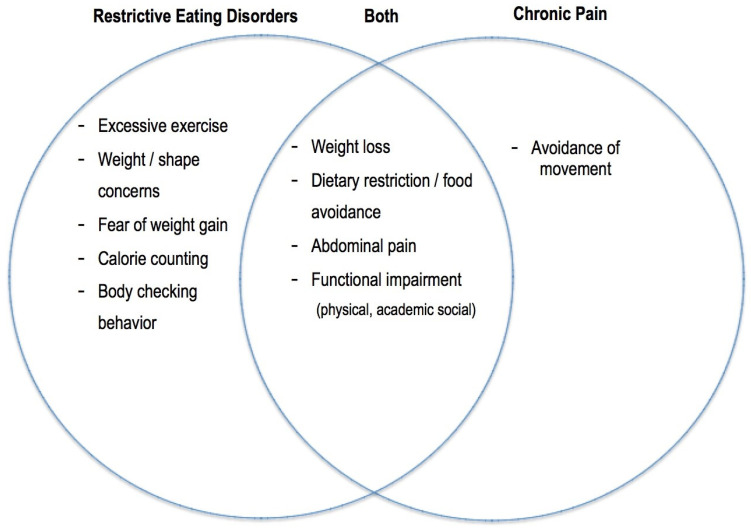
Differentiating and shared symptoms between restrictive eating disorders and chronic pain.

As discussed above, there is no existing protocol or screening tool specifically aimed at identifying comorbid CFAP and restrictive EDs. The integration of standardized guidelines would aid in improvement in the detection and diagnosis of comorbid restrictive EDs and CFAP. Sim and colleagues [13] highlighted behavioral signs of eating disorders for medical professionals to consider when seeing a patient with chronic pain, including CFAP, who may *also* have an eating disorder. Behaviors such as tracking calories, having a calorie “goal” without input from a medical professional, having unrealistic or unhealthy goal weights, excessive exercise, and body checking are often present in the context of an eating disorder but are unlikely to be present in adolescents with CFAP alone. Similarly, an intense fear of weight gain and significant body dissatisfaction are often reported by adolescents with eating disorders [62] but would not likely be experienced by adolescents who have CFAP without a comorbid eating disorder. Future directions for the assessment of eating disorders drawing from these guidelines might involve highlighting key behaviors and symptoms that could differentiate a patient with CFAP alone from a patient with a restrictive ED, with or without CFAP (See Figure 1). 

An assessment approach aimed at distinguishing between CFAP and eating disorder comorbidities might also involve the use of hypothetical questions, such as: “If you were not in pain, would you avoid eating ___?” or “How might your diet be different if you did not experience abdominal pain?”. Responses to such questions could provide insight into the specific factors driving the patient’s dietary restriction. Additionally, patient responses to recommendations for increasing their caloric intake in response to weight loss could be informative [22]. It would be expected that a patient with CFAP might respond to conversations around increased food intake with anxiety due to fear of pain, but it would be unexpected for a patient with CFAP to respond with anxiety due to fear of weight gain. In the case of the latter, the response might be reflective of a potential eating disorder.

In summary, both a careful medical history and an assessment of psychological and behavioral factors are critical to the assessment of a comorbid restrictive ED and CFAP. Discussion among interdisciplinary clinicians (e.g., physician, psychologist, nutritionist), the adolescent, and parents surrounding the adolescent’s psychological functioning may aid in the identification of an ED pathology. Indeed, research posits that a primary focus on medical symptoms may delay the identification and treatment of eating disorders [13]. Therefore, a thorough assessment protocol including the consideration of psychological symptoms may be needed to detect restrictive EDs in patients with atypical presentations, such as youth with CFAP.

## 6. Current Treatment Practices for CFAP and Restrictive EDs

### 6.1. Treatment for CFAP

Treatment for CFAP often includes a combination of dietary changes and pharmaceutical treatments (e.g., laxatives, probiotics) [2,48]. Effective psychological interventions include cognitive behavioral therapy (CBT) and relaxation strategies [63,64]. 

It has been demonstrated in the literature that interdisciplinary pain treatment (IPT) is an efficacious treatment approach for children and adolescents with chronic pain conditions, such as CFAP [50]. IPT involves coordinated care among pediatric psychologists, pediatricians, and physical therapists [49,50]. Pediatricians might recommend dietary changes, such as a low FODMAP (fermentable oligo-di-mono-saccharides and polyols) diet [65]. Importantly, there is insufficient evidence either for or against the use of a low FODMAP diet in youth with CFAP. Laxatives, probiotics, and antispasmodics are also common pharmaceutical treatment options for certain individuals [2]. Psychological interventions are considered a strong treatment option. Specifically, pediatric psychologists often use cognitive behavioral therapy (CBT) [63,64], relaxation strategies [66], and guided imagery [67] to help patients to cope more effectively with chronic pain. Physical therapy/rehabilitation strategies might include exposure to movement in the presence of pain as well as aiding the patient in setting and achieving functional restoration [66].

### 6.2. Treatment for Restrictive EDs

There are several treatment options and settings for restrictive eating disorders, as well as different levels of care depending upon the patient’s medical status [68,69]. Treatment for restrictive EDs typically consists of medical care (medical stabilization, nutrition counseling, medications) and psychotherapy, with some patients needing to be hospitalized for medical stabilization prior to engaging in partial hospitalization or outpatient psychotherapy [70]. Evidence-based therapies often include family-based treatment (FBT [71]) and/or individual cognitive-behavioral therapy (CBT) approaches [72,73]. FBT and CBT can be delivered in across treatment settings [71,74,75,76].

## 7. Recommendations for Treatment of CFAP and Restrictive EDs

When it comes to treatment options for *comorbid* CFAP and restrictive EDs, no standardized, evidence-based interventions exist. However, there are some consistencies across treatment modalities and techniques. For example, in both EDs and CFAP, dietary restriction should be avoided. CBT is an evidence-based treatment for both pediatric chronic pain and restrictive EDs [63,64,71,74,75,76]. Pain-focused CBT includes teaching effective pain coping strategies and reducing negative thinking patterns [63,64]. CBT for restrictive EDs (i.e., CBT-E) includes identifying maintenance factors; reducing negative thoughts around shape, weight, and food; and reducing negative behaviors (e.g., dietary restriction, excessive exercise) [77,78]. Given the presence of commonalities across intervention approaches, it certainly seems plausible that a unified, transdiagnostic CBT approach could be helpful for adolescents with comorbid CFAP and ED symptoms. Such an intervention could take into consideration thoughts, feelings (e.g., anxiety), and behaviors (e.g., avoidance of certain foods) unique to CFAP and restrictive EDs, as well as those relevant to both conditions.

For adolescents with comorbid CFAP and restrictive EDs, maintenance factors need to be determined. One possibility is fear of pain. Based on the fear-avoidance model of pain, [79] as well as classical conditioning and chronic pain, if an individual associates food with pain, they are more likely to respond with avoidance behavior [80]. Pain avoidance through food refusal or dietary restriction could also maintain restrictive eating. Targeting a fear of pain within a CBT framework might involve graded exposure in therapy or at home (delivered by caregivers) to help the adolescent challenge their cognitions related to fear of pain or specific foods in a way that allows them to address the avoidance behavior in a stepwise manner [81]. For example, if an adolescent avoids eating rice because they associate doing so with abdominal pain, graded exposure might involve first (1) touching the rice, (2) preparing the rice, (3) smelling the rice, (4) putting rice in their mouth, and finally (5) swallowing the rice [79,80,81,82].

Another CBT strategy that might be effective in the treatment of comorbid CFAP and restrictive EDs is self-monitoring, which can be conducted using a thought log [83]. Thought logs could be used to help increase awareness (in the present moment) so that the adolescent learns to notice, interrupt, and challenge automatic thoughts (and subsequently, maladaptive behaviors) related to pain avoidance, food restriction, exercise, etc.

### 7.1. Special Considerations: Dietary Changes

There are special considerations to make when treating adolescents with comorbid CFAP and restrictive EDs. CFAP patients are asked to make dietary changes in order to avoid foods that trigger gastrointestinal distress, such as adopting a low FODMAP diet [65,84]. However, within the context of restrictive EDs, clinicians may need to approach recommendations for dietary changes with caution. When possible, clinicians should avoid prescribing strict elimination diets to patients with comorbid EDs until the care team determines whether the patient is capable of doing so in a healthy manner. 

If dietary changes are made in an effort to manage CFAP symptoms, these should be monitored closely by the entire interdisciplinary care team (i.e., physician, psychologist, dietitian) so as not to reinforce restrictive eating patterns, maladaptive eating behaviors, or prevent the patient from overcoming psychological distress when faced with foods deemed “unhealthy” or pain-inducing [22]. Exposure to a variety of foods is, thus, key regardless of whether avoidance behavior is associated with pain and/or weight gain [13,22]. Thus, strong communication among eating disorder and chronic pain clinicians throughout treatment is critical to improve the accuracy and speed at which problematic behaviors, such as avoidance, are identified and treated. 

### 7.2. Importance of Interdisciplinary Care

Pediatric chronic pain conditions (such as CFAP) and eating disorders involve biological, psychological, and social factors, which require thoughtful, critical consideration for optimal assessment, diagnosis, and treatment [13]. Increasing communication and reducing disconnect between eating disorder and pain clinicians could improve the accuracy and speed at which eating disorders and pain comorbidities are identified and treated. Multidisciplinary teams often carry out diagnostic and treatment procedures for restrictive eating disorders and CFAP in youth [85,86]. In multidisciplinary care, various disciplines communicate with one another regarding patients. However, in some clinical settings, evaluation, case conceptualization, and diagnosis occur independently of other disciplines, despite recommendations advocating for an interdisciplinary approach. Mounting research evidence suggests that restrictive eating disorders and CFAP could be diagnosed and treated effectively with an interdisciplinary team [87,88]. Interdisciplinary care involves the weaving together of perspectives and methods from more than one discipline in the evaluation, conceptualization, and diagnosis of patients [87,88,89]. Although the interdisciplinary models for restrictive eating disorders possess strengths, such as integrated evaluations and treatment-synthesized recommendations from clinical-nutritional, endocrino-gynecological, and psychiatric providers [88], the models do not promote the involvement of pain specialists in assessment and/or diagnostic procedures. Similarly, in models of interdisciplinary care for pediatric chronic pain, the involvement of eating disorder providers is not explicitly recommended [50,87]. Increasing communication and reducing the disconnect between eating disorder and pain specialists could improve the accuracy and speed at which eating disorder and pain comorbidities are identified and treated.

Although no studies have examined the effectiveness of multidisciplinary care or interdisciplinary care in the diagnosis and treatment of *both* restrictive eating disorder and CFAP, it is plausible that interdisciplinary care may be more effective when it comes to diagnosis and treatment given the complexity of the comorbid presentation, as well as the overlap in symptoms [13,49] between the two conditions. Future research should involve a randomized control trial in order to determine the efficacy of an interdisciplinary approach compared to multidisciplinary approach. Moreover, from a clinical perspective, providers should work towards an interdisciplinary model of treatment in order to provide more fluid and comprehensive assessment, diagnostic, and treatment services. This could improve the detection, diagnosis, and treatment of comorbid CFAP and restrictive eating disorders.

## 8. Conclusions

In the limited literature on the identification and diagnosis of restrictive eating disorders among youth with CFAP, screening and assessment methods are inconsistent. Atypical presentations of eating disorders, such as restrictive EDs and comorbid abdominal pain, could obscure psychopathology. Indeed, eating disorders in adolescents with chronic pain take longer to be identified. Delayed intervention has grave implications for treatment and recovery [13,26]. Future research efforts should aim to (1) address the diagnostic practices and protocols utilized in pediatric settings most likely to see adolescents presenting with symptoms of CFAP and/or EDs (i.e., adolescent medicine, gastroenterology, chronic pain, primary care and family medicine clinics) and (2) improve communication among medical and mental health providers in order to promote the rapid and effective diagnosis and treatment of comorbid CFAP and restrictive Eds. 

## Data Availability

Not applicable.
